# Surveillance and the global fight against cholera: Setting priorities and tracking progress

**DOI:** 10.1016/j.vaccine.2019.06.037

**Published:** 2020-02-29

**Authors:** Andrew S. Azman, Sean M. Moore, Justin Lessler

**Affiliations:** a Department of Epidemiology, Johns Hopkins Bloomberg School of Public Health, Baltimore, USA; b Department of Biological Sciences, University of Notre Dame, USA; c Eck Institute for Global Health, University of Notre Dame, USA

**Keywords:** Vibrio cholerae, Cholera, Elimination policy, Vaccine, Surveillance

Prior to 1961, there were six known cholera pandemics. In each case, after 6–24 years of global spread cholera appears to have died out [1]. In contrast, the current seventh pandemic that emerged around 1961 continues to cause significant disease globally. Recent outbreaks occurring in the wake of conflict and natural disasters, notably those in Haiti, Yemen and South Sudan, have highlighted the human toll of cholera. Annual cholera seasons throughout South Asia and sub-Saharan Africa have become the norm. At the same time, new tools for cholera control are increasingly available, in particular, effective low-cost oral cholera vaccines (OCVs) [2]. These developments have catalyzed global cholera control efforts including the call for an international effort to end cholera as a public health threat by 2030 [3].

Past experience with smallpox, polio and malaria has taught us that such disease elimination efforts not only call for expanded control activities, but also substantial improvements in surveillance – from its essential role in allocating resources to how case finding, and case definitions, must change as we come closer to reaching our goals. However, cholera’s specific epidemiology poses unique challenges that must be addressed to move forward. Here, building on lessons from historical efforts, we propose three pillars for success in setting priorities and measuring progress in the fight against cholera: consistent reporting across time and space, appropriate indicators and matching interventions to populations.

## What makes cholera different?

Fighting cholera and documenting progress comes with a number of cholera-specific challenges due to its diverse transmission dynamics, the lack of highly cholera-specific symptoms, that laboratory confirmation has little bearing on appropriate treatment and that no intervention alone can sustainably control cholera.

Unlike malaria and measles, cholera exhibits a spectrum of transmission dynamics ranging from epidemic, where countries report zero cases in some years and large outbreaks in other years, to endemic, where cases occur regularly with seasonal peaks. The substantial lull periods of cases in many countries combined with non-specific cholera symptoms, make it difficult for surveillance to maintain focus on consistently tracking cholera with high specificity. Furthermore, reporting cholera has historically been thought to affect tourism and exports within a country, leading to underreporting or no reporting at all.

Unlike smallpox and polio, there is no ‘silver bullet’ to protect people and halt transmission. While OCVs have moderate medium-term effectiveness [2], any serious effort to control cholera must rely on water and sanitation infrastructure improvements and hygiene-related behavior change. These infrastructure and behavior changes are not simple and require large sustained investments.

## The need for enhanced cholera surveillance

Assessing progress requires that we quantify the burden of cholera. However, only a small fraction of suspected cholera cases are laboratory confirmed. The World Health Organization (WHO) does not recommend exhaustive confirmation, in part because confirmation rarely influences clinical care [4]. The WHO’s suspected cholera case definition in areas without a confirmed outbreak is ‘*a patient ≥ 5 years [who] develops severe dehydration or dies from acute watery diarrhea*,’ whereas during outbreaks, all acute watery diarrhea patients ≥ 5 years are counted [4]. While case definitions vary by country, they are usually similar to the WHO’s, meaning even well-functioning surveillance systems have low specificity for detecting true cholera cases. This may be particularly true in highly endemic settings where incidence is concentrated in children. Reporting of suspected cholera alone, especially when case definitions vary from place to place, can result in a distorted picture of the true geographic distribution of cholera, both due to differences in the circulation of other pathogens and simple differences in reporting. Without a more refined and standardized look at cholera burden, we may incorrectly allocate resources and have a false understanding of our success in fighting cholera. While exhaustive confirmation of suspected cases would provide ideal data, it is not realistic. Instead, serious efforts should be made in standardizing case definitions within and across countries and ensuring that they are appropriately applied.

The true proportion of suspected cases that would be confirmed cases varies from place-to-place and season-to-season, thus location-specific sentinel surveillance with routine confirmation of suspected cases is key to estimating the true number of cases from suspected case counts. We suggest this is done in endemic and epidemic settings during both low and high-transmission periods and across the urban/rural gradient. These estimates can then be combined with routinely collected acute watery diarrhea incidence data from a larger geographic area (ideally nationally) to produce high resolution estimates of cholera burden. Understanding the etiology and seasonality of other diarrheal pathogens across all ages can help further refine this picture.

## Pillar 1: Consistent reporting across time and space

Consistent reporting of cholera cases across time and space is a critical first step in estimating the burden of cholera. Currently, countries are asked to report suspected cholera cases each year to the WHO, although reports tend to be aggregated annually and over the country [5]. Furthermore, some countries under report or simply do not report cases, including priority countries in the fight against cholera like Bangladesh and Ethiopia [5]. Only after all countries start reporting cholera cases can we truly track progress in global cholera control.

The WHO should ask that countries provide data disaggregated by week and district (or equivalent) and explicit case definitions should be provided for each location. As with other diseases like malaria, an assessment of the quality of reporting can be made using simple indicators like the proportion of health facilities reporting each week [6].

## Pillar 2: Appropriate indicators for cholera

Given the dynamic nature of cholera, especially in areas that exhibit more epidemic dynamics, assessing incidence or mortality rates in a single year can be misleading. For example, Lusaka, Zambia [7], had annual cholera outbreaks until 2012 with no confirmed cholera cases reported until 2016. If only annual incidence were considered in 2013–2015, we would have declared success. While far from perfect, we believe that incidence rates averaging over 5 years, perhaps longer, should be used as one primary measure of cholera burden.

Time-averaged incidence rates can be slow to change, meaning annual updates may provide little new information on progress. We propose the development of location-specific hypothesis tests that would yield the probability of data from that current year (or N-years) being indicative of a true reduction in the risk of cholera at a specific location, as compared to expected year-to-year random incidence variation. As more locations report zero cases each year, this could evolve into using statistical methods to estimate the probability that cholera is truly eliminated in a location. This type of location-specific definition can guard against false declarations of elimination, which may occur frequently if a simple definition (e.g., no cases for 3 consecutive years) is used across all areas, irrespective of the local cholera epidemiology.

Estimating cholera mortality can be extremely challenging given that in some places many cases lack access to care [8]. Without more active surveillance, we will have to rely on location-specific models for cholera mortality that may have substantial uncertainty. While these estimates may be used in tracking progress, it may be more practical to focus on mortality in clinics, an indicator for quality of care. At the least, community and facility deaths should be tracked separately.

Other potential indicators include the outbreak recurrence frequency, proportion of months with active cholera transmission, the age distribution of cases, and measures of the geographic expansion of an outbreak after a confirmed case. Global efforts to map appropriate indicators of water and sanitation use [9] may also prove particularly useful for assessing risk when reported cholera cases are low.

## Pillar 3: Identifying and prioritizing high-risk populations

**Fig. 1:**
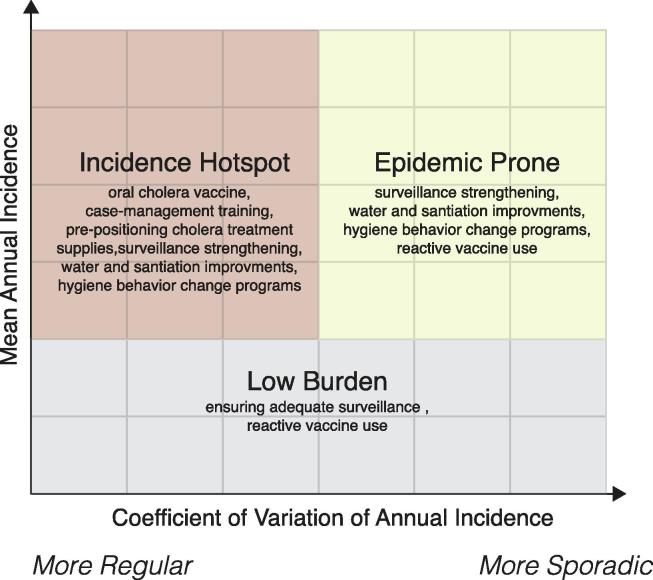
An example of cholera classification system based on mean annual incidence (y-axis) and coefficient of variation in annual incidence (x-axis). Small text underneath classifications highlights the potential measures that may be considered for areas falling into each category.

Focusing cholera control efforts on entire countries is not feasible, thus identification of high-risk populations is essential for guiding the optimal intervention targets and types. Ongoing efforts to map historical mean incidence rates of reported cholera cases may provide one avenue for identifying those at-risk [10]. However, incidence characterizes only one dimension of risk/burden. One possible simple classification system to help match the cholera epidemiology to interventions could be based on considering the (weighted) mean annual incidence rate and variability in annual incidence rates for sub-country administrative units [10]. This two-dimensional system can help us classify areas as being (1) cholera incidence hotspots, (2) cholera epidemic-prone and (3) low burden (Fig. 1).

While the numerical cutoffs for indicators used to classify areas may vary by settings, the classifications can be used to prioritize interventions. For example, in incidence hotspots, both short-term and long-term approaches are needed, combining the use of OCV and preparations for appropriate case management with long-term behavior change and infrastructure programs. In epidemic-prone areas, preemptive OCV use may be less appropriate given the year-to-year variation in risk and the short-term vaccine protection, unless the periodicity of risk is well understood. However, as these areas may be prone to large but infrequent outbreaks, plans for rapid reactive OCV use should be put in place. In lower burden settings, while the mean annual incidence may be low, similar investments in infrastructure and behavior change can ensure they do not act as gateways of transmission to other neighboring areas and that they do not transition into epidemic prone or incidence hotspots, perhaps after a complex emergency. This system is simple enough to apply across all cholera-prone countries although it neglects many potentially important local features of cholera epidemiology. Thus, location-specific epidemiologic knowledge should supplement any broad intervention prioritization system.

## Success for cholera means success everywhere

While the fights against smallpox and polio had broad impacts in building international health systems and local expertise, the tools used in each primarily impact a single disease. Success in fighting cholera would have broad impacts on health, far beyond those specific to cholera. Though an essential tool for control, OCV protection is impermanent and the bacteria may persist in the environment. Hence, the only long-term solution is access to safe water and improved sanitation and behavior change. Sustained improvements here, though focused on cholera, would allow people to enjoy broad health benefits, such as access to clean drinking water. Cholera is then not just a goal itself, but a rallying point for a broader fight against water-related pathogens and an essential indicator of our progress. That every previous pandemic came to an end tells us the fight against cholera can be successful, and success would be a general public health win, not simply related to cholera.

## Funding

This work was supported by the Bill & Melinda Gates Foundation, Seattle, WA [OPP1171700], [OPP1191944] and National Institutes of Health (R01 AI135115).

## Declaration of Competing Interest

We declare no conflicts of interest.
